# Participation of Short-Chain Fatty Acids and Their Receptors in Gut Inflammation and Colon Cancer

**DOI:** 10.3389/fphys.2021.662739

**Published:** 2021-04-08

**Authors:** María Daniella Carretta, John Quiroga, Rodrigo López, María Angélica Hidalgo, Rafael Agustín Burgos

**Affiliations:** Laboratory of Inflammation Pharmacology, Faculty of Veterinary Science, Institute of Pharmacology and Morphophysiology, Universidad Austral de Chile, Valdivia, Chile

**Keywords:** short-chain fatty acid, FFAR2, FFAR3, HCAR2, colon cancer, inflammatory bowel disease

## Abstract

Short-chain fatty acids (SCFAs) are the main metabolites produced by the bacterial fermentation of dietary fiber, and they play a critical role in the maintenance of intestinal health. SCFAs are also essential for modulating different processes, and they have anti-inflammatory properties and immunomodulatory effects. As the inflammatory process predisposes the development of cancer and promotes all stages of tumorigenesis, an antitumor effect has also been associated with SCFAs. This is strongly supported by epidemiological studies showing that a diet rich in fiber is linked to a reduced risk of colon cancer and has significant clinical benefits in patients with inflammatory bowel disease (IBD). SCFAs may signal through the metabolite-sensing G protein-coupled receptors free fatty acid receptor 3 [FFAR3 or G protein-coupled receptor 41 (GPR41)], FFAR2 (GPR43), and GPR109A (also known as hydroxycarboxylic acid receptor 2 or HCAR2) expressed in the gut epithelium and immune cells. This review summarizes the existing knowledge regarding the SCFA-mediated suppression of inflammation and carcinogenesis in IBD and colon cancer.

## Introduction

The gut microbiota contributes to the development and function of the host metabolic and immune system (Belkaid and Hand, 2014; Cani, 2014). An imbalance of the microbiota, known as dysbiosis, is produced by several factors, including the “Western diet,” low dietary fiber consumption, and extrinsic stressors, such as antibiotic exposure that can lead to chronic inflammation and metabolic dysfunction (Lobionda et al., 2019). This state has been associated with increasing numbers of diseases, including inflammatory bowel disease, obesity, and cancer (Zuo and Ng, 2018; Vivarelli et al., 2019; Lee et al., 2020). For this, the interplay between the gut microbiome and host cells has gained attention in recent years.

Key candidates underlying this link are the short-chain fatty acids (SCFAs)—the major microbial metabolites from the bacterial fermentation of dietary fiber produced in the intestine. The principal SCFAs are acetic acid (C2), propionic acid (C3), and butyric acid (C4), and they constitute 95% of the total SCFAs in mammals (Cummings et al., 1987; Bergman, 1990). The SCFA concentrations can reach levels as high as 70–100 mM in the gut lumen with the ratio of acetate, propionate, and butyrate in the colon being 60:25:15 (Sellin, 1999). Following their production, SCFAs can be absorbed by passive diffusion or can be transported into the cell by substrate transporters, like monocarboxylate transporter 1 (MCT1; encoded by SLC16A1), which is coupled to H^+^ transport, and the sodium-coupled monocarboxylate transporter 1 (SMCT1; encoded by SLC5A8), which is likely to be the main transporters (Ritzhaupt et al., 1998; Miyauchi et al., 2004). Once they are absorbed by colonocytes, they enter the citric acid cycle in the mitochondria to generate energy (McNeil, 1984). SCFAs that are not metabolized by the colonocytes are transported through portal circulation, where concentrations of 260 μM acetate, 30 μM propionate, and 30 μM butyrate have been found (Bloemen et al., 2009). In the liver, SCFAs are used as energy substrates for hepatocytes by acetyl-CoA synthetase (Schönfeld and Wojtczak, 2016) and acetate and butyrate are substrates for the synthesis of cholesterol and long-chain fatty acids (Boets et al., 2017; Kindt et al., 2018). Also, SCFAs absorbed in the sigmoid colon and rectal region can also reach systemic circulation directly through the inferior vena cava (Canfora et al., 2015). Total SFCA concentration in the peripheral blood is between 80 and 180 μM, with acetate predominating, and fiber-rich diets result in increased SFCA plasma levels (Offermanns, 2014; Canfora et al., 2015). These data indicate that SCFAs produced by the microbiota are transported from the intestinal lumen into the blood compartment of the host and are taken up by organs where they act as substrates or signaling molecules that regulate several cellular processes and systemic effects (Den Besten et al., 2013; McNabney and Henagan, 2017; Chen et al., 2020a; Maruta and Yamashita, 2020). These effects are mediated mainly by two pathways: the inhibition of histone deacetylases (HDACs) and the activation of cell surface receptors (Licciardi et al., 2011; Ganapathy et al., 2013).

HDACs are a conserved family of proteins that have four classes depending on their sequence homology to *Saccharomyces cerevisiae* HDACs (Kim and Bae, 2011). These enzymes catalyze the removal of an acyl group on a histone bound to chromatin resulting in a tight complex and repression of gene expression (Davie, 2003). SCFAs are non-competitive inhibitors of HDACs and specifically inhibit the activity of HDAC1 and HDAC3 which belongs to class I HDACs (Davie, 2003; Thangaraju et al., 2006; Singh et al., 2010). This effect favors histone acetylation that produces an open and more transcriptionally active chromatin resulting in increased transcription of genes but, in the case of butyrate, results in both induction and repression of specific genes (Sivaprakasam et al., 2016a). Among the SCFAs, butyrate is the most potent inhibitor of HDACs with ~80% inhibitory effciency. In experiments using nuclear extracts from HT-29 human colon carcinoma, an IC_50_ = 0.09 mM has been calculated (Waldecker et al., 2008; Kasubuchi et al., 2015). Other SCFAs such as propionate were less potent with a maximum inhibitory efficiently of ~60%, and various studies indicate that acetate has no HDAC inhibitory activity (Kiefer et al., 2006; Singh et al., 2010; Kasubuchi et al., 2015). The inhibition of HDAC activity by SFCAs (mainly butyrate) finally results in the induction of the transcription of specific genes contributing to intestinal homeostasis and apoptosis and/or cell cycle arrest in colon cancer cells (Kim and Bae, 2011; Fung et al., 2012a).

The second mechanism for SCFA effects is signaling through G protein-coupled receptors (GPCRs). The major GPCRs activated by SCFAs are the free fatty acid receptor 3 [FFAR3 or G protein-coupled receptor 41 (GPR41)], FFAR2 (GPR43), and G protein-coupled receptor 109A, GPR109A (also known as hydroxycarboxylic acid receptor 2 or HCAR2) (Offermanns, 2014; Kimura et al., 2020). Therefore, there is a growing interest in manipulating and modulating the activity of these receptors as potential therapeutic targets in disease areas, such as metabolic disorders, cancer, and inflammatory conditions of the lower gut. Great advances have occurred in recent years, and there now exist several orthosteric and allosteric agonists and antagonists of the FFAR2 receptor. For this, we will focus specially on the currently available pharmacological tools for the study of FFAR2. We will also summarize the current knowledge of the implications of FFAR2 and HCAR2 on gut inflammation and colon cancer.

## Short-Chain Fatty Acid Receptors

SCFAs are widely known to mediate several physiological and pathophysiological effects in the intestines and other tissues; however, the mechanisms by which they carried out these actions only began to be understood in 2003. Previously, FFAR2 and FFAR3 were discovered in a cluster as a group of intronless sequences located in close proximity to CD22 on human chromosome 19q13.1 (Sawzdargo et al., 1997). They contain seven transmembrane domains, characteristic of GPCRs, along with other common features and were classed as orphan GPCRs (Sawzdargo et al., 1997). Then, they were cloned and demonstrated to be receptors for SCFAs by three independent groups in 2003 (Brown et al., 2003; Le Poul et al., 2003; Nilsson et al., 2003).

### FFAR2/3 and HCAR2 Pharmacology, Signaling, and Expression

The potency of SCFAs on FFAR2 and FFAR3 is low, between ranges of high micromolar to millimolar concentrations. Their potency is dependent on the size of the SCFA chains. The order of potency for FFAR2 was consistent between various studies, with acetate (C2) and propionate (C3) equipotent followed by butyrate (C4), valerate (C5), and formate (C1) (Brown et al., 2003; Le Poul et al., 2003; Nilsson et al., 2003). The FFAR3 receptor shows a stronger affinity to the longer SCFAs C4 = C3 = C5 > C2 = C1 (Brown et al., 2003; Le Poul et al., 2003). Species-dependent variations have been detected; e.g., for the mouse receptors, C2 is equipotent in activating FFAR2 and FFAR3 (Hudson et al., 2012a), while the order of potency is C4 > C3 > C2 for FFAR2 in bovines (Hudson et al., 2012b).

Although FFAR2 and FFAR3 share the same endogenous ligands, they are coupled to different G proteins. FFAR3 initiates signaling mainly coupled to Gαi/o proteins, whereas FFAR2 couples to both the Gαi/o and Gαq pathways (Stoddart et al., 2008). FFAR2 and FFAR3 activation via Gαi/o inhibits adenylyl cyclase and, subsequently, decreases the levels of cyclic AMP (cAMP). The two receptors activate the ERK cascade (Le Poul et al., 2003; Vinolo et al., 2011a); however, in the case of FFAR2, the activation of ERK1/2 is mainly via Src proteins and that for FFAR3 is via PI3K (Seljeset and Siehler, 2012).

The activation of FFAR2 via Gαq results in a rise in intracellular Ca^2+^ concentrations (Brown et al., 2003; Le Poul et al., 2003). This is mediated by phospholipase C (PLC) activation that induces the hydrolysis of phosphatidylinositol 4,5-biphosphate (PIP_2_) into diacylglycerol and inositol 1,4,5-triphosphate (IP_3_). IP_3_, in turn, activates the IP_3_ receptors placed at the endoplasmic reticulum, and this finally leads to the release of cytosolic Ca^2+^ (Kimura et al., 2020). The contribution of the double coupling of FFAR2 in physiological processes is not yet fully resolved. Additionally, the FFAR2 receptor also engages an alternative signaling pathway mediated by β-arrestins-2, producing anti-inflammatory effects through the inhibition of nuclear transcription factor kappa B (NF-κB) (Gao et al., 2004; Lee et al., 2013).

FFAR2 and FFAR3 expression has been identified throughout the gastrointestinal tract. FFAR3 is highly expressed in the ileum of mice (Samuel et al., 2008), while the highest expression of FFAR2 was observed in the rat colon (Dass et al., 2007). In enteroendocrine L cells, FFAR2 is highly expressed and SFCA mediates the release of peptide YY (PYY) and glucagon-like peptide 1 (GLP-1) from the lower gut of both mice and humans (Tolhurst et al., 2012; Chambers et al., 2015). FFAR3 is also expressed in the human colon (enterocytes and enteroendocrine cells) but less compared to FFAR2 (Tazoe et al., 2009). In adipose tissue, FFAR2 is expressed in white adipocytes and also in resident macrophages and is involved in the reduction in lipolysis and fat accumulation (Bolognini et al., 2016, 2021). In pancreatic beta cells, both receptors are expressed and regulate glucose-stimulated insulin secretion (GSIS) (Tang et al., 2015; Pingitore et al., 2019). It has been observed that FFAR2 differentially regulates GSIS via Gαq/11 and Gαi/o resulting in either potentiation or inhibition of GSIS, respectively (Priyadarshini et al., 2015). FFAR3 receptor is mostly linked to negative regulation of insulin secretion (Priyadarshini and Layden, 2015; Veprik et al., 2016). In the peripheral nervous system, FFAR3 activation increased the heart rate and energy expenditure (Kimura et al., 2011).

The expression of FFAR2 is abundant in immune cells, where it is involved in a variety of cellular processes. This receptor is expressed in polymorphonuclear cells, monocytes, eosinophils, basophils, and particularly high in neutrophils (Brown et al., 2003; Le Poul et al., 2003; Nilsson et al., 2003). Several studies have shown that FFAR2 mediates the chemotactic effects of SCFAs in neutrophils (Le Poul et al., 2003; Maslowski et al., 2009; Sina et al., 2009; Vinolo et al., 2011b). Neutrophil activation by SCFAs results in the formation of reactive oxygen species (ROS) (Stringer et al., 1996; Nakao et al., 1998; Maslowski et al., 2009) likely mediated by FFAR2. Negligible amounts of SCFA are present in peripheral blood (Cummings et al., 1987; Bergman,1990), with acetate the only SCFA present in concentrations high enough to induce FFAR2-dependent signaling (Le Poul et al., 2003) (e.g., EC_50_ of calcium flux ~100 μM) and support the role of SCFA in the neutrophils chemotaxis FFAR2-dependent (Vinolo et al., 2011b). However, Maslowski et al. showed that the neutrophils in FFAR2 knockout (KO) mice decreased ROS release and phagocytic activity, which was increased by SCFAs in wild-type (WT) mice (Maslowski et al., 2009). These authors and Senga et al. also demonstrated FFAR2 expression in murine bone marrow and spleens suggesting a possible role of this receptor in the differentiation of immune cells (Senga et al., 2003). FFAR2 is highly expressed on colonic epithelial cells and T regulatory (Treg) cells (Smith et al., 2013), and research demonstrated that the modulation of colonic Tregs by SCFAs via FFAR2 is critical to maintaining intestinal immune homeostasis (Kespohl et al., 2017). In this way, higher concentrations of SCFA in the digestive tract suggest a key role of FFAR2 and FFAR3 on the gut in both physiological and pathophysiological conditions.

The HCAR2 receptor (also known as GPR109A in humans and PUMA-G in mice) belongs to a family of receptors that also includes GPR81 (HCAR1) and GPR109B (HCAR3), which share significant sequence homology and respond to endogenous hydroxy-carboxylic acid metabolites (Offermanns, 2014). This receptor was initially identified as the receptor of the anti-dyslipidemic and anti-atherogenic drug nicotinic acid (Soga et al., 2003; Tunaru et al., 2003; Wise et al., 2003). Nicotinic acid (niacin) is a member of water-soluble vitamins that shows an agonist effect on HCAR2 with a EC_50_ in the range 0.06–0.25 μM (Wise et al., 2003); however, the endogenous levels of nicotinic acid are too low to significantly impact receptor activity. The ketone body β-hydroxybutyrate (Taggart et al., 2005) and the FFA butyrate (Thangaraju et al., 2009) were recognized as physiological agonists for the receptor. Butyrate activates HCAR2 only with low affinity (EC_50_ value of 0.7 mM) (Taggart et al., 2005), and propionate and acetate do not activate the receptor. However, pentanoate (C5), hexanoate (C6), heptanoate (C7), and octanoate (C8) can induce [^35^S] GTPγS binding to membranes from CHO cells expressing HCAR2 with an EC_50_ in range of 0.13–0.73 mM (Taggart et al., 2005).

The ligand binding of HCAR2 indicates that HCAR2 is pertussis toxin-sensitive, and therefore this receptor family couples to Gαi/o-type G proteins (Tunaru et al., 2003; Wise et al., 2003). For this, HCAR2 activation results in the diminished activity of adenylate cyclase, which leads to the inhibition of cAMP production as demonstrated in adipocytes (Soga et al., 2003; Tunaru et al., 2003; Wise et al., 2003). In neutrophils and macrophages, the activation of HCAR2 by nicotinic acid resulted in a transient increase in Ca^2+^ (Benyó et al., 2005; Kostylina et al., 2008). HCAR2 agonists also induced ERK1/2 activation in human Langerhans cells (Richman et al., 2007) and in the human epidermoid carcinoma cell line, A431 (Tang et al., 2006). The internalization process of HCAR2 is via the clathrin-coated pit pathway, and the agonist-induced internalization is regulated by arrestin-3 and G-protein-coupled receptor kinase-2 (GRK2) (Li et al., 2010). This receptor also forms homodimers with itself and heterodimers with HCA3 in a process that is not regulated by ligand binding and occurs early during the receptor biosynthesis (Mandrika et al., 2010).

HCAR2 is predominantly expressed in human adipocytes and functions as a metabolic sensor, suppressing lipolysis during periods of food starvation to avoid the excessive degradation of triglycerides (Tunaru et al., 2003; Taggart et al., 2005). The HCAR2 receptor is widely expressed in various immune cells, such as neutrophils (Kostylina et al., 2008), dendritic cells, and macrophages (Schaub et al., 2001). In the intestinal tract, the expression is limited to the lumen-facing apical membrane of intestinal and colonic epithelial cells where butyrate is found in concentrations able to activate the receptor (Thangaraju et al., 2009; Sivaprakasam et al., 2017). However, in germ-free mice, the expression of HCAR2 in these cells was reduced but returned to normal levels when the intestinal tract was colonized with bacteria, indicating that the expression of this receptor is induced by the gut microbiota (Cresci et al., 2010).

### FFAR2/3 Agonists

The development of selective agonists and antagonists of FFA receptor has been necessary for a major understanding of the multiple effects of SCFA and to investigate the potential therapeutic effects of modulating FFA receptor activity. This has been undoubtedly challenging since the potency of these molecules at both FFAR2 and FFAR3 is low given their small size, which results in a low binding energy (Schmidt et al., 2011; Smith, 2012). These receptors also display overlapping tissue expression with a similar pharmacology between them responding to the same SCFAs that are coupled to Gi/o proteins. The use of KO mice that could help the physiological characterization has also shown limitations. For example, the expression of FFAR2 was also reduced in FFAR3 KO mice (Zaibi et al., 2010), thereby masking the purpose of the study. Despite these obstacles, an increasing number of agonists and antagonists have been reported that act at the orthosteric and allosteric sites of FFAR2.

#### FFAR2/3 Synthetic Allosteric Agonists

One of the first reported synthetic phenylacetamide derivative activators of FFAR2 was 4-chloro-α-(1-methylethyl)-N-2-thiazolylbenzeneacetamide, also called 4-CMTB or phenylacetamide 1. This ligand revealed an additional binding pocket in FFAR2, distinct from that for the endogenously produced SCFAs. In fact, mutagenic studies confirmed that extracellular loop 2 plays an important role in the FFAR2 allosteric agonist effect of 4-CMTB (Smith et al., 2011). AZ1729 is a direct allosteric FFAR2 agonist and a positive allosteric modulator that increases the activity of SCFAs, such as propionate in Gi-mediated pathways but not at those transduced by Gq/11 and, therefore, considered “Gi” -biased allosteric agonists (Bolognini et al., 2016). For FFAR3, the only ligand characterized as an allosteric agonist and positive allosteric modulator of SCFAs is 4-(furan-2-yl)-2-methyl-5-oxo-N-(o-tolyl)-1,4,5,6,7,8-hexahydroquinoline-3-carboxamide (FHQC), activating Gi and reducing the cAMP inhibition of human FFAR3, being inactive at FFAR2 (Hudson et al., 2014). An analog of this compound, AR420626, also an allosteric agonist at FFAR3, was used to demonstrate a role for FFAR3 in GLP-1 release from murine colonic crypt cultures (Engelstoft et al., 2013) and was shown to suppress neurogenic motility in rat proximal colons (Kaji et al., 2017).

Another phenylacetamide compound that acts as an allosteric agonist of FFAR2 is [(S)-2-(4-chlorophenyl)-3,3-dimethyl-N-(5-phenylthiazol-2-yl)butamide (CFMB/Cmp58)], with ~750-fold higher potency than the SCFAs on the rat FFAR2 (Wang et al., 2010; Christiansen et al., 2018), and is also an allosteric FFAR2 modulator (Lind et al., 2020). In neutrophils, AZ1729 and Cmp58 did not increase intracellular calcium but potentiated the propionate response. AZ1729 also turned Cmp58 into a potent activator of the superoxide-generating neutrophil NADPH oxidase. AZ1729/Cmp58 together activates neutrophils to produce O^2−^ and induce a selective FFAR2 desensibilization, permitting that the orthosteric agonist propionate could still induce a transient rise in intracellular Ca^2+^ (Lind et al., 2020).

#### FFAR2/3 Synthetic Orthosteric Agonists

In 2013, Hudson et al. described the first potent and selective FFAR2 orthosteric agonists, called “compound 1” [3-benzyl-4-(cyclopropyl-(4-(2,5-dichlorophenyl)thiazol-2-yl)amino)-4-oxobutanoic acid], and “compound 2” [(*R*)-3-(cyclopentylmethyl)-4-(cyclopropyl-(4-(2,6-dichlorophenyl)thiazol-2-yl)amino)-4-oxobutanoic acid], which possessed a selective orthosteric agonist effect at human FFAR2 and demonstrated key aspects of ligand interaction within the binding site of FFAR2. These compounds activated signaling via both the Gi- and Gq/11-mediated pathways and promoted interactions between the receptor and β-arrestin-2 in human FFAR2 while not affecting the activity of FFAR1, FFAR3, or FFAR4 (Hudson et al., 2013).

In addition, the mutation of either Arg 5.39 or Arg 7.35 blocked responses to both compounds, confirming interactions within the orthosteric binding pocket (Hudson et al., 2013). However, a number of residues were identified outside the core binding pocket for SCFAs that, when mutated, reduced the affinity/potency of both compounds. The replacement of the extracellular loop 2 from FFAR2 with the comparable sequence from FFAR3 substantially reduced the potency of the Compound-1 and Compound-2; however, this mutation only slightly affected the potency of the SCFA C3 (Milligan et al., 2017).

Another specific FFAR2 agonist was developed, (2S, R5)-5-(2-chlorophenyl)-1-1(2′-methoxy-[1,1′-biphenyl]-4carbonyl)pyrrolidine-2-carboxylic acid (compound 3), without any interaction with FFAR1/FFAR3 or possessing effects on a broad range of other drug targets (Forbes et al., 2015). More recently, a potent thiazolidine FFAR2 orthosteric agonist, called TUG-1375, was shown to be effective to induce human neutrophil chemotaxis and reduce lipolysis in murine adipocytes and, moreover, possessed high solubility and favorable pharmacokinetic properties being an interesting tool compound for the further exploration of FFAR2 (Hansen et al., 2018).

### FFAR2/3 Antagonists

The search for antagonists for FFAR2 has also been of great interest to define its specific functions compared to FFAR3. The first antagonist described was (S)-3-(2-(3-chlorophenyl) acetamido)-4-(4-(trifluoromethyl)phenyl) butanoic acid (CATPB), which is a highly selective FFAR2 orthosteric antagonist (p*K*_*i*_ = 7.87 ± 0.08) (Sergeev et al., 2016) but with no significant affinity at mFFAR2 (Hudson et al., 2013). GLPG0974 (4-[[1-(benzo[b]thiophene-3-carbonyl)-2-methylazetidine-2-carbonyl]-(3chlorobenzyl)amino]butyric acid) revealed almost identical affinity (p*K*_*i*_ = 7.88 ± 0.08) (Sergeev et al., 2016) being a complement displaced by the SCFA C3 and CATPB (Milligan et al., 2017).

CATPB also potentiated forskolin-induced cAMP levels, suggesting that this compound also behaves like an inverse agonist (Park et al., 2016). BTI-A-404 [4-[4-(dimethylamino)phenyl]-N-(3,5-dimethylphenyl)-6-methyl-2-oxo-1,2,3,4-tetrahydro-5-pyrimidinecarboxamide] and BTI-A-292, [4-[4-(dimethylamino)phenyl]-N-(4,5-dimethylphenyl)-6-methyl-2-oxo-1,2,3,4-tetrahydro-5-pyrimidinecarboxamide] are two novel orthosteric antagonists of human FFAR2 with inverse agonist properties (Park et al., 2016; Milligan et al., 2017). FFAR3 antagonist AR399519 (abbreviated also as AR19) was demonstrated to selectively inhibit FFAR3 in stably transfected HEK293 cells (Engelstoft et al., 2013).

## The Role of Short-Chain Fatty Acids and their Receptors in Intestinal Inflammation

Inflammatory bowel disease (IBD) is a term that includes conditions with uncontrolled manifestations of inflammation, both intra- and extra-intestinally, such as ulcerative colitis (UC) and Crohn's disease (CD). It is a heterogeneous disorder associated with genetically predisposed individuals but also with environmental factors, like western life habits (Sobczak et al., 2014; Ananthakrishnan, 2015). These conditions considerably affect the quality of life of patients, with a varied series of symptoms including diarrhea, vomiting, fever, abdominal pain, anemia, and weight loss (Sairenji et al., 2017).

Alteration of the intestinal microbiota in patients with IBD compared to healthy individuals was shown to be involved in the pathogenesis of this disease (Mylonaki et al., 2005). IBD patients also showed reduced levels of SCFAs likely due to a reduced level of SCFA-producing bacteria in the intestinal mucosa (Kumari et al., 2013; Wang et al., 2014; Agus et al., 2016). Regarding inflammation, the dysbiosis promotes the production of proinflammatory cytokines, such as IFN-γ, IL-17, TNF-α, or IL-1β, causing epithelial damage and intestinal symptoms by aggravating the inflammatory process (Venegas et al., 2019).

NF-κB was identified as one of the key regulators in this type of cytokines, and its activation is markedly induced in IBD patients (Atreya et al., 2008). A better understanding of the causes and mechanisms involved in IBD is still required, and, until now, no cure for the disease was available, only symptomatic treatments, such as anti-inflammatory drugs and immunomodulators (Na and Moon, 2019). Other strategies have been in development over the last decade, such as fecal microbiota transplantation; however, the safety is still in doubt (Na and Moon, 2019).

The role of FFAR2 in regulating the intestinal inflammatory response was first described by two research groups who determined that it plays a critical role in the recruitment of neutrophils during intestinal inflammation. However, contradictory results were obtained in FFAR2 KO mouse models. Maslowski et al. (2009) demonstrated that the stimulation of FFAR2 by SCFAs was necessary for a normal resolution of certain inflammatory responses because FFAR2 KO mice showed exacerbated or unresolved inflammation in models of colitis induced by dextran sulfate sodium (DSS) and trinitrobenzenesulfonic acid (TNBS). In germ-free mice, these authors also showed that experimental colitis was aggravated and was reverted with acetate supplementation. These results indicated an anti-inflammatory role for FFAR2 (Maslowski et al., 2009). On the contrary, a study by Sina et al. (2009) demonstrated that FFAR2 KO mice showed diminished intestinal migration of neutrophils but were protected against inflammatory tissue destruction in DSS-induced colitis (Sina et al., 2009). Neutrophils play an important role in the maintenance of intestinal homeostasis and the pathogenesis of IBD. In these pathologies, neutrophil and mononuclear cell migration into the lamina propria and epithelial layer mediates the protection against pathogens through the recognition and elimination of antigens that cross the epithelial barrier as well as cytokines (Venegas et al., 2019). Sina et al. also reported the involvement of p38 MAPK in the chemotactic activity of immune cells. They showed a significant difference in the p38 phosphorylation in neutrophils from WT mice compared with FFAR2 KO mice in DSS-induced colitis (Sina et al., 2009).

Supporting the anti-inflammatory role of FFAR2, in 2013 Masui et al. showed that DSS-induced colitis was exacerbated in FFAR2 KO mice through the increase in proinflammatory cytokines, such as TNF-α and IL-17, and through the decrease in anti-inflammatory cytokine IL-10 in the colonic mucosa. They also treated the mice with 150 mM acetate in their drinking water and observed a marked improvement of the disease indices in the WT mice but no effect on the FFAR2 KO mice (Masui et al., 2013). Other authors also confirmed this data treating WT mice with an FFAR2 agonist, and they observed a reduction in DSS-induced colitis compared with controls, indicating that FFAR2 activation plays a key role in the protection of intestinal inflammation (Agus et al., 2016).

**Figure 1:**
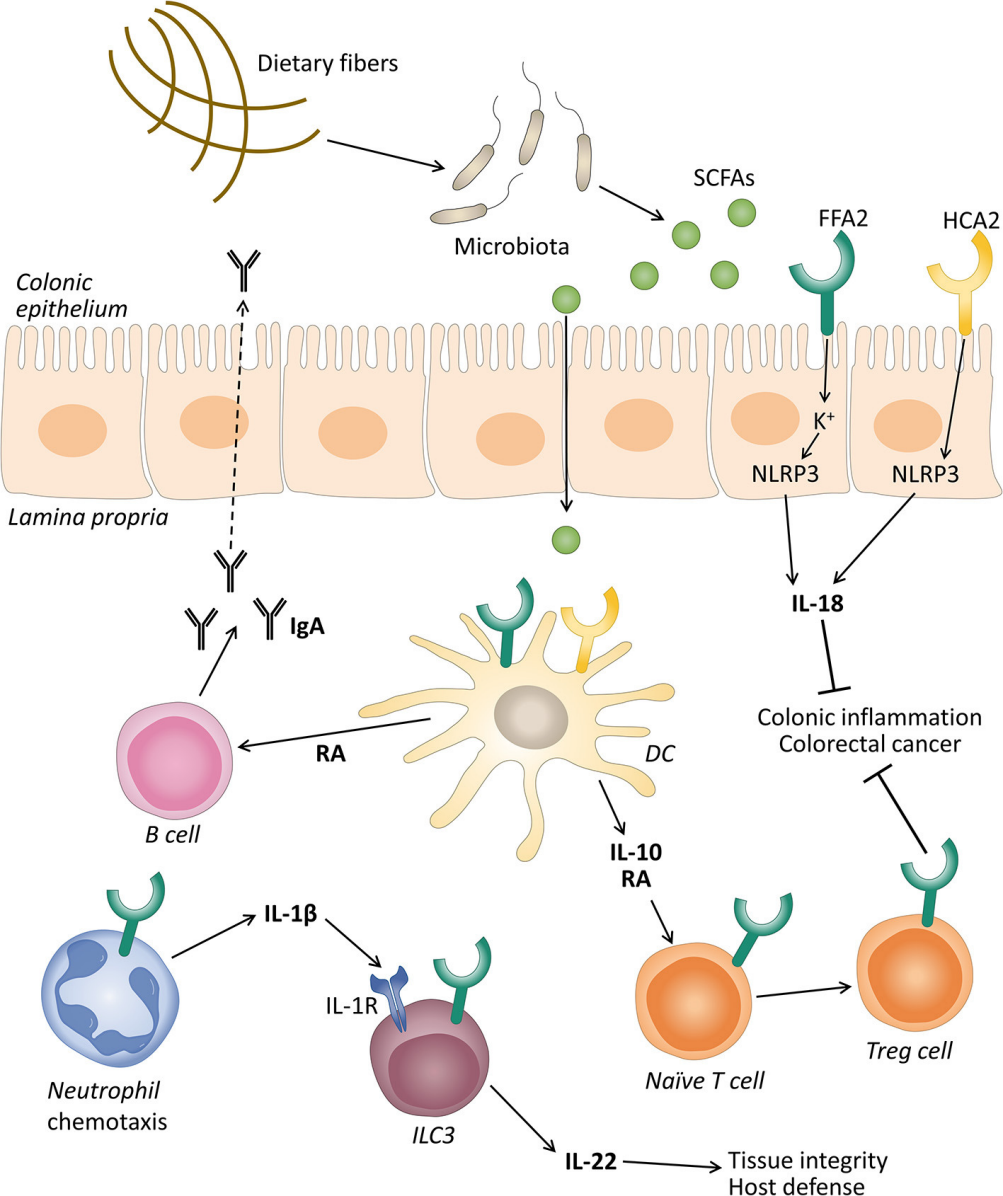
The role of free fatty acid receptor 2 (FFAR2) and hydroxycarboxylic acid receptor 2 (HCAR2) in gut homeostasis. Gut microbiota ferment dietary fibers into short-chain fatty acids (SCFAs), and these can activate their receptors expressed on intestinal epithelial cells and immune cells like neutrophils, dendritic cells (DC), and innate lymphoid cells 3 (ILC3). The activation of FFAR2 and HCAR2 in intestinal epithelial and DC promotes the secretion of interleukin (IL)-18 via inflammasome activation, and IL-10, respectively. IL-10 in turn promotes the differentiation and proliferation of T regulatory (Treg) cells that together with IL-18 protect against conditions leading to colonic inflammation and colorectal cancer. SCFAs through FFAR2 induce neutrophil chemotaxis to inflammatory sites and production of IL-1β. IL-1β engages an IL-1 receptor (IL-1R) on ILC3, promoting IL-22 production that will contribute to tissue integrity and host defense. SCFAs can also stimulate the intestinal immunoglobulin A (IgA) secretion by B cells, induced by retinoic acid (RA) production, although this mechanism is still debated.

The inflammasome pathway and production of the cytokine IL-18 have been reported as the molecular mechanisms that promote gut epithelial integrity, repair, and intestinal homeostasis (Zaki et al., 2010; Elinav et al., 2011). Macia et al. demonstrated that SCFAs were able to stimulate the efflux and hyperpolarization of K^+^ by interacting with FFAR2 and HCAR2 on intestinal epithelial cells, which resulted in an increase in NLRP3 inflammasome activation and the secretion of IL-18 (Figure 1). They proposed that low-fiber feeding and the, consequently, insufficient SCFA levels reduced the effective inflammasome activation through GPCRs (Macia et al., 2015). Recent studies demonstrated that acetate reduced inflammasome activation via FFAR2 in a Ca^2+^-dependent manner. In addition, acetate activated soluble adenylyl cyclase, promoted NLRP3 inflammasome ubiquitination by PKA, and ultimately induced NLRP3 degradation through autophagy (Xu et al., 2019).

Various studies demonstrated that SCFAs promoted the development and function of intestinal Treg cells (Figure 1) (Arpaia et al., 2013; Smith et al., 2013). These cells are considered essential in the induction of the peripheral tolerance to self- and foreign antigens (Fujio et al., 2010). Defects in the number and quality of these cells leads to the development of intestinal inflammation in various animal models (Rubtsov et al., 2008; Huber et al., 2011). These cells produce a large amount of IL-10 which plays a central role in controlling inflammatory processes (Rubtsov et al., 2008; Fujio et al., 2010). Smith et al. showed that oral administration of SCFAs increased the number of colonic Treg cells by stimulating their proliferation (Smith et al., 2013). The mechanisms for the conversion of naive T cells into Tregs by FFAR2 and HCAR2 agonists involves the production of IL-10 and aldehyde dehydrogenase 1a (Aldh1a) by intestinal dendritic cells and macrophages (Coombes et al., 2007; Sun et al., 2007; Singh et al., 2014).

Immunoglobulin A (IgA) responses play a crucial role in maintaining a mutual relationship with commensal bacteria and limiting the access of numerous microorganisms and mucosal antigens at the mucosal surface (Corthésy, 2013). In this regard, research demonstrated that SCFAs promote intestinal IgA responses (Kim et al., 2016), and this role has been attributed in part to the FFAR2 receptor (Wu et al., 2017). Wu et al. reported that FFAR2 KO mice showed decreased production of intestinal IgA compared with WT mice. Acetate also increased intestinal IgA in WT mice in a T-cell-independent manner (Wu et al., 2017). The mechanism proposed was that the activation of FFAR2 by acetate on dendritic cells leads to the expression of the protein Aldh1a2 which converts vitamin A into its metabolite retinoic acid (RA), which induces B cell IgA production (Figure 1) (Wu et al., 2017). However, recent data excluded the implication of FFAR2 in butyrate-induced IgA production in the mouse large intestine. These authors proposed that this effect was mediated by FFAR3 and HCAR2 and the inhibition of HDACs (Isobe et al., 2020). Therefore, the exact mechanism to explain the IgA production induced by SFCAs is still under debate.

Innate lymphoid cells 3 (ILC3s) are effectors of innate immunity that play an important role in the induction, regulation, and resolution of inflammatory responses (Klose and Artis, 2016). Colonic ILC3s in mice express FFAR2, and natural and synthetic ligands promote their proliferation and the production of IL-22, contributing to the host defense against DSS-induced colonic injury and *Citrobacter rodentium* infection in mice (Chun et al., 2019). Current data showed that acetate, via FFAR2, enhanced the expression of the IL-1 receptor (IL-1R), which facilitated IL-22 production in murine ILC3s upon stimulation with IL-1β (Fachi et al., 2020). These authors also revealed that, in neutrophils, acetate-FFAR2 signaling facilitated the initial recruitment of neutrophils and inflammasome activation and augmented IL-1β secretion in response to *Clostridium difficile*, indicating a cross talk between the FFAR2 receptor expression in neutrophils and ILC3s (Fachi et al., 2020) (Figure 1).

The HCAR2 receptor activation by butyrate was also implicated in colonic health mainly through anti-inflammatory effects. The deletion of this receptor in mice accelerated the progression of colonic inflammation (Singh et al., 2014) and produced an altered expression of genes related to multiple inflammatory signaling pathways in intestinal colonic cells (Zimmerman et al., 2012). Additionally, researchers observed that HCAR2 was essential for butyrate-mediated induction of the anti-inflammatory cytokine IL-18 in the colonic epithelium (Singh et al., 2014). The decreased levels of IL-18 were associated with colitis in NLRP6 KO mice (Elinav et al., 2011). IL-18 can promote the production of antimicrobial peptides and can control the gut microbiota composition (Levy et al., 2015). Other studies observed that the NLRP3-mediated inflammasome plays a significant role in IL-18 secretion through HCAR2, thus promoting gut epithelial integrity (Figure 1) (Macia et al., 2015). The anti-inflammatory properties of HCAR2 are also caused by inducing IL-10 and Aldh1a expression in dendritic cells and macrophages, which facilitated the conversion of naïve T cells into Treg cells (Figure 1) (Singh et al., 2014). HCAR2 activation has also been implicated in the regulation of ILC3s. An *in vitro* study showed that butyrate stimulation decreased the number of ILC3s and the IL-22 production compared with a vehicle control in terminal ileal Peyer's patches (Kim et al., 2017). According to these data, the HCAR2 agonists niacin also reduced the number of ILC3s in the colon and suppressed IL-23 production by dendritic cells (Bhatt et al., 2018). However, this is still controversial and under study, as the influence of the SCFAs on the frequency of ILC3s in the gut may depend on the different subpopulations of ILC3s across different anatomical sites (Zhou and Sonnenberg, 2020).

All these data establish a critical role for FFAR2 and HCAR2 in the regulation of multiple cytokines relevant to the etiology of IBD.

## Role of SCFA and their Receptors in Colorectal Cancer

Colorectal cancer (CRC) is one of the most common malignant tumors worldwide with almost 900,000 deaths annually (Dekker et al., 2019). Common symptoms of CRC include changes in bowel habits like diarrhea or constipation, rectal bleeding, abdominal pain, and loss of weight (Thanikachalam and Khan, 2019). Disturbances in the normal microbial balance caused by environmental changes are frequently observed in CRC patients (Gagnière et al., 2016; Alhinai et al., 2019). Several bacterial species (e.g., *Fusobacterium nucleatum, Streptococcus bovis, Bacteroides fragilis, Escherichia coli, Enterococcus faecalis*, and *Peptostreptococcus anaerobius*) have been strongly related with colorectal carcinogenesis (Cheng et al., 2020; Janney et al., 2020). In addition, an increased risk of CRC was associated with the decreased production of SCFAs (Gomes et al., 2018). FFAR2, an important regulator of colonic inflammation, was proposed as a tumor suppressor; however, the exact role is still under investigation (Cosín-Roger et al., 2020).

SCFAs play essential roles in the colonic heath and integrity, regulating the colonic mobility, blood flow, and gastrointestinal pH (Den Besten et al., 2013). Several studies have shown the antitumor effects of SCFAs with a major participation of butyrate. For example, butyrate exhibits anti-tumorigenic functions by inhibiting proliferation or inducing apoptosis in a variety of human CRC cells at physiological concentrations (Fung et al., 2012a,b) and several other cancer cells, such as breast cancer cells (Semaan et al., 2020), bladder cancer cells (Wang et al., 2020), and lung cancer cells (Xiao et al., 2020). This function has been attributed to butyrate's ability to alter gene transcription by inhibiting histone deacetylase activity (Fung et al., 2012a); however, the participation of FFA receptors has also been postulated. FFAR2 is present in the human colon and rat distal ileum and colon (Karaki et al., 2006, 2008; Tazoe et al., 2008).

Tang et al. reported a dramatic reduction in FFAR2 expression in human colon malignant adenocarcinoma tissues. The re-expression and activation of this receptor in CRC cells inhibited proliferation and induced apoptosis and cell cycle arrest, providing evidence that FFAR2 functions as a tumor suppressor (Tang et al., 2011). Other authors showed that *FFAR2*(-/-) mice were hypersusceptible to the development of intestinal tumors (Sivaprakasam et al., 2016b). They demonstrated that FFAR2 signaling stimulated the growth of beneficial bacteria, such as *Bifidobacterium*, and decreased the amount of unfavorable gut microbiota, such as *Helicobacter hepaticus* and *Prevotellaceae*, promoting a healthy gut (Sivaprakasam et al., 2016b).

Additional studies supported the idea of a protective role for FFAR2 in a variety of mouse models of colon carcinogenesis (Macia et al., 2015; Kim et al., 2018). The most recent findings revealed that FFAR2 is an important mediator for HDAC inhibition induced by butyrate, indicating an epigenetic tumor suppressor role for FFAR2 and blocking colon cancer progression. These authors also demonstrated that FFAR2 deficiency promoted colon tumorigenesis through overexpression of the cAMP-PKA-CREB and Wnt pathways and increased HDAC protein expression (Pan et al., 2017, 2018). As of now, the search is still on to understand the underlying molecular mechanisms elicit by FFAR2.

New data provided evidence that, when FFAR2 and FFAR3 expression was reduced, increased levels of glucose transporter 1 (GLUT1), with the subsequent increase in glucose uptake, was observed in the HCT116 CRC cell line (Al Mahri et al., 2020). This finding could be relevant since the overexpression of GLUT1 was suggested as a negative prognostic biomarker in CRC and an indicator of aggressive clinical features in CRC (Yang et al., 2017). These authors also excluded the role of PKA-mediated cAMP signaling on cell proliferation and glucose uptake; therefore, alternate pathways require further study (Yang et al., 2017).

Other mechanisms have recently been proposed by Lavoie et al., in which a loss of FFAR2 promoted colon tumorigenesis in mice by reducing the gut barrier integrity, increasing the tumor bacterial load, and altering the dendritic cell expression of IL-27 and the phenotypes and functions of CD8^+^ T cells in tumors (Lavoie et al., 2020). Other authors, using *Clostridium butyricum* (*C. butyricum*), a butyrate-producing probiotic, reported that this bacterium inhibited intestinal tumor development by modulating Wnt/β-catenin signaling (Chen et al., 2020b). Aberrant Wnt signaling was implicated in CRC progression (Antas et al., 2019; Gay et al., 2019). The siRNA-mediated gene silencing of FFAR2 reduced the anti-proliferative effect of *C. butyricum*, and thus the activation of FFAR2 might be linked to the protective role of this bacteria (Chen et al., 2020b).

In 2009, Thangaraju et al. reported that HCAR2 was expressed on the luminal plasma membrane of the epithelial cells in the colon and small intestine. In addition, they observed that this receptor was silenced in colon cancer in humans, in a mouse model of colon cancer, and in various colon cancer cell lines as a result of DNA methylation (Thangaraju et al., 2009). The re-expression of HCAR2 in colon cancer cell lines and its activation by butyrate abolished NF-κB activation and induced apoptosis (Thangaraju et al., 2009). These findings opened an alternative mechanism, apart from HDACs in the induction of apoptosis by butyrate. Further studies revealed that HCAR2 functions as a suppressor of carcinogenesis in the colon. The HCAR2 knockdown in mice accelerated the progression of colonic inflammation and colon cancer in multiple experimental model systems (Singh et al., 2014; Macia et al., 2015). Bardhan et al. reported a novel mechanism underlying the regulation of HCAR2 expression in colon cancer cells. They proposed that the host immune system might use IFNγ to counteract DNA methylation-mediated HCAR2 silencing as a mechanism to suppress tumor development (Bardhan et al., 2015).

## Conclusions

In this review, we focused on the beneficial effects of SCFAs and their receptors in gut homeostasis. These molecules exert a plethora of biological effects through their receptor and other mechanisms. Efforts to achieve a comprehensive understanding of the role of these receptors in some pathophysiological situations continue. However, one of the obstacles is the overlapping expression of FFAR2 and FFAR3 in the same tissues or types of cells and the fact that they have the same endogenous ligands. For this, the search of new specific agonists and antagonists for FFAR2, FFAR3, and HCAR2 as potential targets to achieve the maintenance of intestinal health is critical. GLPG0974, a selective antagonist of the human FFAR2, is considered a potential candidate molecule to inhibit neutrophil activation and subsequent recruitment in the gut.

The first clinical trials using this antagonist observed that GLPG0974 potently inhibited the acetate-induced expression of activated CD11b on human neutrophils, *in vitro* as well as *ex vivo*. This molecule did not cause side effects and was well-tolerated (Namour et al., 2016). However, one of the inconveniences with this antagonist is that it did not show affinity at mouse or rat FFAR2, making preclinical testing difficult in these species (Sergeev et al., 2016). Another strategy used in animal models and in UC patients is the modulation of the microbiota using prebiotics and probiotics that can enrich the microbiome diversity; however, there is limited evidence indicating clinical improvement.

The expression of the SCFA receptors in various immune cells, such as ILC3s, opens new scenarios in the immunomodulation of SCFAs in the gut. ILC3s have emerged as master regulators of intestinal health, and their responses are altered in the guts of patients with inflammatory and metabolic diseases. Therefore, further investigations are necessary to better understand the impact of microbiome-derived metabolites and their receptors in the possible interplay between ILC3s and the other immune cells that can shape the intestinal immune system.

## Author Contributions

MC and RB drafted the initial manuscript. MH, RL, and JQ provided critical feedback. JQ designed the figure. All authors contributed to the article and approved the submitted version.

## Conflict of Interest

The authors declare that the research was conducted in the absence of any commercial or financial relationships that could be construed as a potential conflict of interest.
